# A Comparison of Stocking Methods for Pasture-Based Growing-Finishing Pig Production Systems

**DOI:** 10.3390/ani10101885

**Published:** 2020-10-15

**Authors:** Silvana Pietrosemoli, James T. Green, Maria Jesús Villamide

**Affiliations:** 1 Department of Animal Science, College of Agriculture and Life Sciences, North Carolina State University, Raleigh, NC 27695, USA; 2 Departamento de Producción Agraria, E.T.S.I. Agronómica, Alimentaria y de Biosistemas, Universidad Politécnica de Madrid, 28040 Madrid, Spain; mariajesus.villamide@upm.es; 3 Department of Crop and Soil Sciences, College of Agriculture and Life Sciences, North Carolina State University, Raleigh, NC 27695, USA; jim_green@ncsu.edu

**Keywords:** growing-finishing pigs, pasture-based pig production, stocking methods, tall fescue (*schedonorus arundinaceus*), ground cover, soil nutrients

## Abstract

**Simple Summary:**

A sustainable pasture-based production system should provide benefits to the vegetation, soil and animals while providing means of economic support for the household that operates the system. If best management practices are implemented, this would allow the development of resilience for the pasture system and ameliorate the natural resources for present and future generations. Pasture-based pig production represents a production alternative for small scale or limited-resources farmers, offering them the possibility to brand their products. The implementation of best management practices would allow managers to reach productive and conservation goals. This study was conducted to compare the effects of continuous, rotational and strip-grazing stocking methods for growing-finishing pigs on tall fescue pastures. Stocking methods had effect on soil bulk density and some soil nutrients, vegetative ground cover, animal weight gain and feed use efficiency. The rotational and strip grazing stocking methods offer potential to improve the sustainability of pasture-based pig systems.

**Abstract:**

Two alternative stocking methods (rotational and strip-grazing) were compared to continuous stocking at a stocking rate of 47 pigs ha^−1^ in tall fescue pastures. The research was conducted during two twelve-weeks grazing periods in North Carolina (USA). In total 144 (females and castrated males, 17.5 and 29.1 kg initial body weight) crossbred Yorkshire X Berkshire, Yorkshire/Landrace X Hampshire and Yorkshire/Landrace X Duroc pigs without nose rings were used. Greater soil bulk density and soil concentrations of NO_3_^−^, P, K, Mn, Zn and Cu were observed in paddocks managed continuously, while greater final ground cover (+22%) was recorded in paddocks managed with rotational and strip-grazing stocking methods. No differences were detected in botanical composition of the paddocks. Greater weight gains (+8.5%) were registered for rotationally managed pigs. Feed efficiency was better (+8%) for rotationally than for continuously stocked pigs, while strip-grazed pigs presented intermediate values. The results indicated the potential of both alternative stocking methods to be implemented in sustainable pasture-based pig production systems.

## 1. Introduction

The sustainability of pasture-based animal production systems has social, environmental and economic implications. Grasslands provide low cost feed for livestock, mainly ruminants, becoming the basis for production and livelihoods in many rural areas of the world [1]. Sustainable grazing systems are grounded on the principles of improving the cycle of nutrients of grassland ecosystems, enhancing the vegetation, increasing the biodiversity and improving the performance of grazing animals [2]. The adoption of one or another management strategy can represent a significant difference on the productivity, environmental impact and consequently the sustainability of a production system [1,3]. Adequate management strategies need to be implemented to fulfill the varied functions and services of grassland ecosystems [2]. According to Motta-Delgado et al., a sustainable pasture-based production system should provide benefits to the vegetation, the soil and the animals, while providing means of economic support for the household. The implementation of best management practices would allow for the development of resilience of the pasture system and conserve and ameliorate the natural resources for present and future generations [1].

Pasture-based pig production represents a production alternative to small scale or limited resource farmers due to its relatively low initial investment requirements and the possibility to brand their products via diversification. It also offers an option for consumers searching for pork perceived as produced in more animal-welfare and environmentally-friendly circumstances [4,5] due to emerging or niche markets, considered a good approximation to sustainable meat production [6]. Paradoxically, the same advantage of pasture-based pig systems that allow the expression of natural behaviors could represent a disadvantage in conditions of mismanagement of the animals. Habits such as foraging, rooting, trampling and selecting dunging areas could cause damage to the ground cover [7,8,9], create bare soil areas [8,10], soil compaction [11], upload nutrients and create nutrients concentration in preferred defecating spots [12,13,14]. In turn, those behaviors can increase the risks of erosion, runoff and nutrient losses.

Pietrosemoli and Green advocate for management of grazing pigs that focus on the main impacts that this animal species could have on pastures [4]. This implies that minimizing ground cover disappearance [13,15,16], decreasing the damage to the soil structure [17,18,19], reducing buildup of soil nutrients [8,15,16,20] and enhancing soil nutrients distribution [9,13,15,16,21] should be taken into consideration when designing the pasture management plan.

The stocking method most frequently used to keep pigs on pastures is the continuous stocking method [22], where pigs have continuous, unrestricted access to a grazing area, usually for the length of a production cycle. This method is often preferred by farmers because of its lower initial investment in water-supplying systems and fencing [23] and minimum labor needs. Some alternatives to continuous stocking such as rotational stocking methods, provide a rest period to forages that allow recovery and regrowth, potentially improving their productivity and persistence [24,25]. European wild boar did not show differences in grazing behavior nor pasture consumption between animals managed in continuous or rotational systems [23].

While there is a profusion of information related to stocking methods for other kinds of pasture-based livestock, to our knowledge there is limited information about how growing-finishing pig stocking methods impact soil properties, vegetation and animal performance. The purpose of this research is to contribute to the generation of knowledge about this subject by evaluating the effect of pig stocking methods on tall fescue pastures. This knowledge would enable improving management practices for pasture-based pig production, thus reducing pollution and enhancing the environment. In this study, two rotational alternative stocking methods (rotational and strip-grazing) were compared to continuous stocking at the same stocking rate in a tall fescue pasture-based pig system.

## 2. Materials and Methods

### 2.1. Study Area

**Figure 1:**
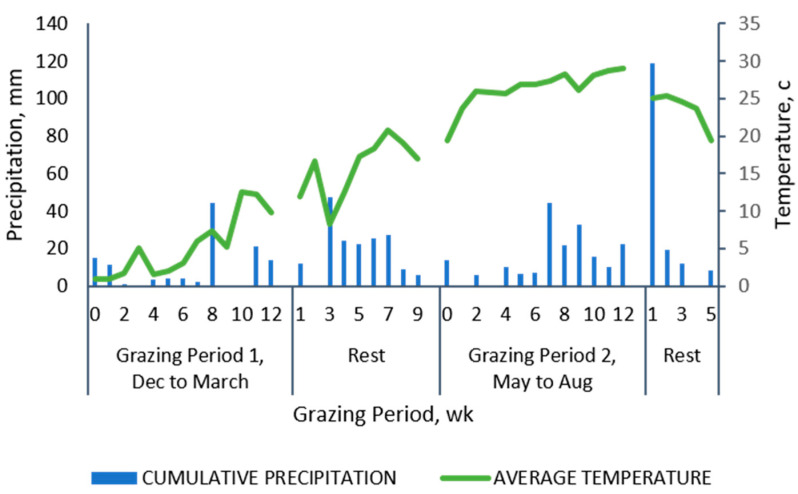
Cumulative precipitation and average temperature during the experimental period. Source: State climate office of North Carolina [27].

The research was conducted during two twelve-week grazing periods (December to March and May to August, with 12 weeks of rest between them), in Goldsboro (35.38291° N, 78.035846° W, 24 m above sea level), North Carolina (USA). The farm is the Cherry Research, Education and Outreach Facility of the Center for Environmental Farming Systems (CEFS). The research site comprised a 1.5 ha tall fescue (*Schedonorus arundinaceus* (Schreb.)) field with no previous history of pastured pigs. The soils were classified as Johns loamy sand (Fine-loamy over sandy or sandy-skeletal, siliceous, semi-active, thermic Aquic Hapludults) with 0% to 2% slope [26]. According to the Trewartha climate classification, the climate is humid subtropical. Considering as a reference data from the last four years (2016–2020), the average precipitation was 1598 mm with a year round distribution and two peaks, one in April–May and the other in September–October. The average annual temperature was 17.3 °C (11.1 to 22.5 °C), being January the coldest month (0.9 °C) and July (32.7 °C) the warmest (NC CRONOS/ECONet Database and personal estimation) [27] (Figure 1).

The 1.5 ha pasture was divided into three blocks which were subdivided into three paddocks each. Due to some irregularities in the field, one of the replicates had slightly different dimensions than the other two (105 m × 48.3 m for replicates 1 and 2 and 93 m × 54.5 m for replicate 3). On average, each paddock measured 1689.8 m^2^. Eight pigs were used in each paddock to a fixed stocking rate equivalent to 47 pigs ha^−1^. This stocking rate was selected based in previous observations at the farm where it was possible to maintain 60% of tall fescue ground cover at the end of the pig growing cycle with stocking rates of 212 m^2^ pig^−1^.

### 2.2. Experimental Design and Treatments

The experimental design was a randomized complete block design, with three field-replicate blocks. Three pig stocking methods were compared during two twelve-weeks grazing periods (Table 1). The stocking methods were randomly distributed to the paddocks in each block. A split-plot arrangement of treatments was employed to analyze the soil related variables, with stocking method as the main plot factor and soil sampling depth (0 to 15 cm or 15 to 30 cm) as the sub-plot factor. For ground cover, the grazing period was considered the main plot and stocking method the subplot, while botanical composition was analyzed as a complete block with the stocking method as the source of variation. Regarding animal related variables, the grazing period was the main plot and stocking method the subplot.

**Figure 2:**
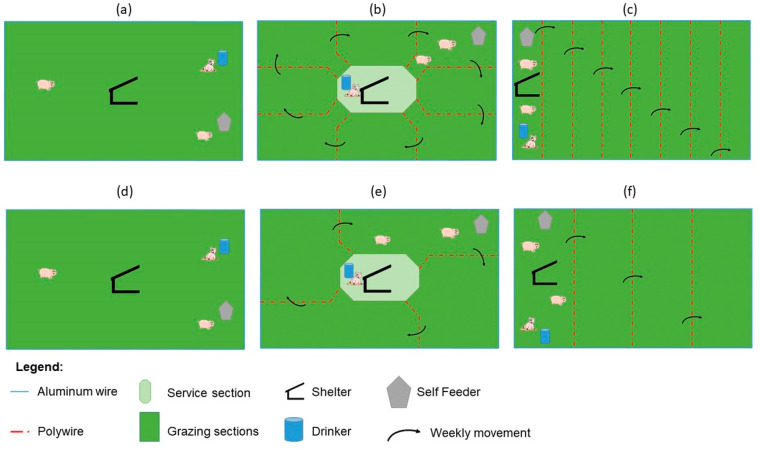
Stocking methods under comparison, weeks 1 to 8: (**a**) Continuous, (**b**) Rotational and (**c**) Strip-grazing. Weeks 9 to 12: (**d**) Continuous, (**e**) Rotational and (**f**) Strip-grazing. Figures (**d**–**f**) represent figures (**a**–**c**) following removal of certain fences and grazed four additional weeks.

**Figure 3:**
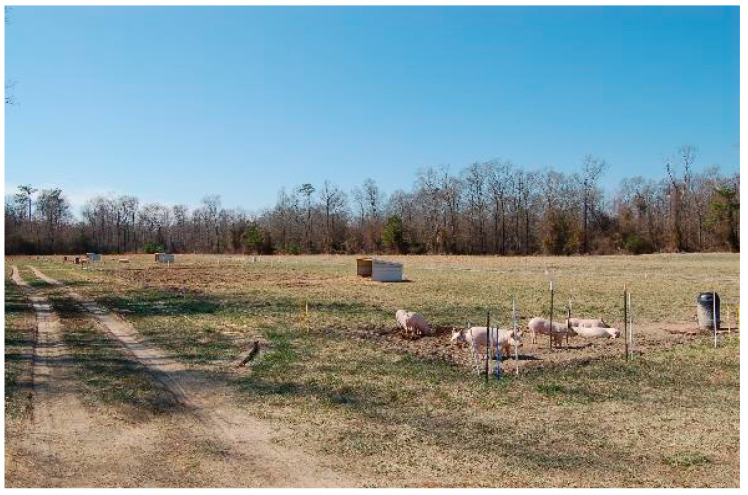
View of a paddock managed under the continuous stocking method during the first grazing period (December to March).

**Figure 4:**
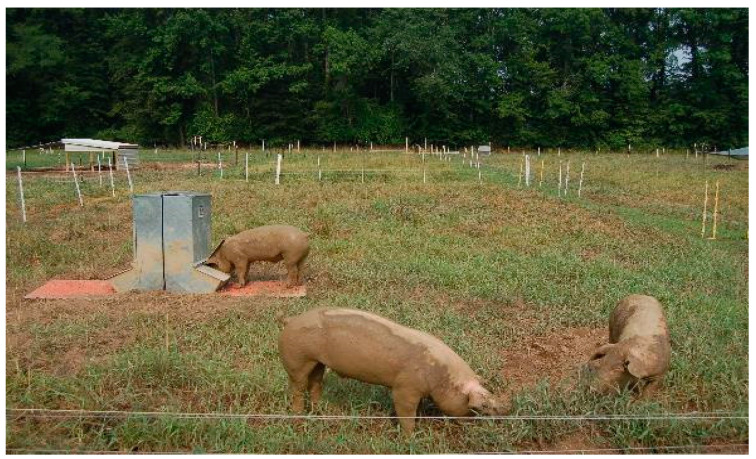
View of a rotationally stocked paddock during the second grazing period (May to August). In front, pigs in the grazing sections. Back left, the service-central section with the shelter and the drinking station.

**Figure 5:**
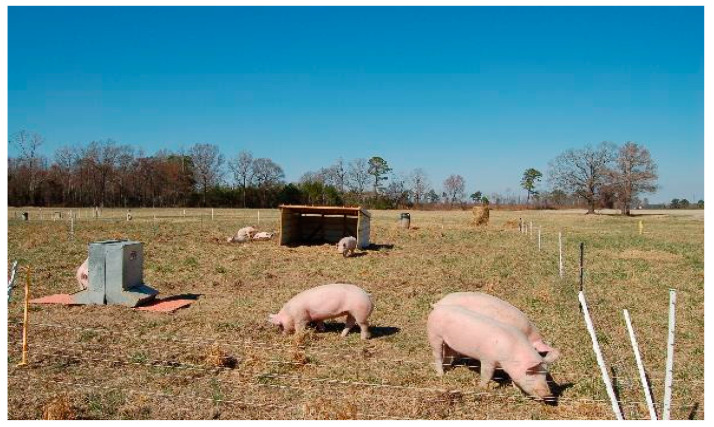
View of a Strip managed paddock during the first grazing period (December to March). In front are two two-space self-feeders, in the center of the strip the shelter, in the back the plastic barrel used as a drinker during winter.

The stocking methods under evaluation (Figure 2) consisted of a continuous stocking system where animals were allowed to roam free in the paddocks during the entire grazing period (Continuous, Figure 3) and two rotational methods. The first rotational method was developed by dividing the paddock into nine equally-sized sub paddocks, including the center area acting as the service area where the shelter and water were placed. Pigs had permanent access to the service area and would graze the other sections on a weekly basis during weeks one to eight, after which they would be allowed to graze two sections per week, following this pattern during week nine to twelve of the grazing period. The feeders were located in the grazing sections and were moved with the animals (Rotational, Figure 4). For the third stocking method, namely strip grazing, the paddock was divided into eight strips. All the service structures (shelters, drinkers and feeders) were moved with the pigs. In this treatment, pigs were allowed to graze one strip per week during weeks one to eight and two strips per week during weeks nine to twelve (Strip grazing, Figure 5). The switch to larger areas following week 8 in the rotational and strip-grazing methods was implemented to fulfill a double function: improving soil nutrients distribution and reducing the stocking density when larger/heavier animals would be grazing the sub-paddocks. A twelve weeks rest period (from March to May) was applied between grazing periods. After being grazed, the sub paddocks for the rotational and strip-grazing treatments were back fenced, thus preventing the animals to have access to them.

Paddocks were considered the experimental unit for most of the variables evaluated, with the exception of animal live weight and weight gain related variables where each animal was studied as the experimental unit. The treatments were kept in the same paddocks for the entire length of the study. 

### 2.3. Animals

The animal-related protocols for this experiment were reviewed and approved by the Institutional Animal Care and Welfare Committee of North Carolina State University (IACUC 09-021-A). A total of 72 not nose-ringed pigs were included in each grazing period. Crossbred Yorkshire X Berkshire female and castrated male pigs (17.5 ± 0.3 kg and 78.6 ± 1.8 kg initial and final body weight, respectively) were used during the first grazing period and Yorkshire/Landrace X Hampshire and Yorkshire/Landrace X Duroc male pigs (29.1 ± 0.8 kg and 91.0 ± 0.9 kg initial and final body weight, respectively) during the second grazing period. The pigs were vaccinated and dewormed prior to moving them to the experimental site. The pigs were ranked according to their live weight and assigned at random to nine groups of eight pigs each, to balance initial total weight among groups. Each group was then assigned at random to the paddocks. Body weights were recorded individually at the beginning and at the end of each grazing period. Animals were weighed at approximately the same time of day in each event, without overnight fasting. Portable corrals and a scale were set-up in the paddocks, rattle paddles, paddle sticks and sorting panels were used to corral the animals. Weight gain was calculated per pig and daily gain was estimated according to the total weight gain and the days of the grazing periods (84 d). Pigs had ad libitum access to water and to a homemade grain mix (corn, soybean, vitamins and minerals) which was formulated following NRC nutrient recommendations for pigs [28]. On average, the grain mixes contained 151.8 g kg^−1^ crude protein, 36.2 g kg^−1^ crude fat and 38.4 g kg^−1^ ash (4.9 g kg^−1^ Ca; 4.9 g kg^−1^ P; 1.6 g kg^−1^ S; 1.6 g kg^−1^ Mg; 0.9 g kg^−1^ Na; 6.4 g kg^−1^ K; 12 mg kg^−1^ Cu; 184 mg kg^−1^ Fe; 32 mg kg^−1^ Mn; and 98.5 mg kg^−1^ Zn) and 2383 kcal kg^−1^ DM (dry matter) of net energy. Feed samples were collected monthly, composited and analyzed per grazing period at the North Carolina Department of Agriculture and Consumer Services NCDA&CS Forage laboratory. Feed disappearance was estimated at the paddock level as feed offered minus feed residues at the end of each grazing period. Feed efficiency was calculated for each grazing period by dividing animal weight gain for each paddock by paddock dry matter feed disappearance. 

### 2.4. Pastures

**Figure 6:**
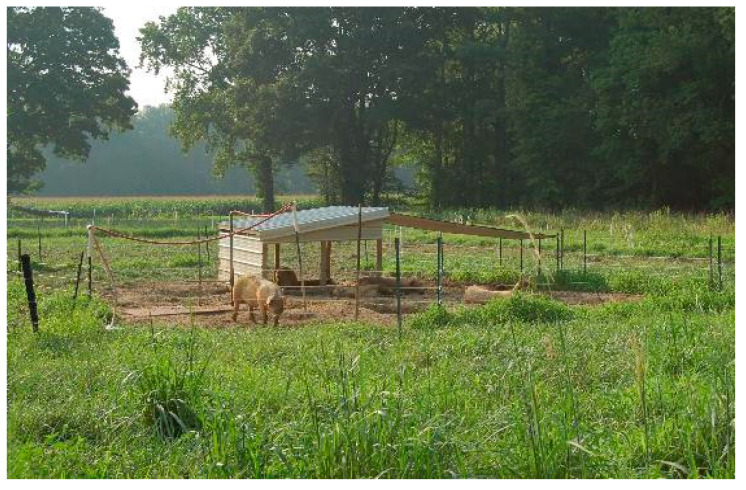
View of the service-central area of a rotationally managed paddock. The portable shelter, the tarp used to provide additional shade and the drinking structure with a hose and two nipple drinkers can be seen. Two walls of the shelter were removed to allow for extra ventilation during summer.

All paddocks were furnished with the same set of equipment: a three-sided wood and zinc-laminate shelter (5.75 m^2^) and two two-space self-feeders. During the first grazing period (winter), plastic water barrels were used to supply water, whereas during the second grazing period two water nipples coupled to metal pipes were employed (Figure 6). During winter, pigs were provided with bedding (hay), while during summer two of the shelter walls were removed to allow for extra ventilation and additional shade was supplied using a tarpaulin (11.2 m^2^). Poly-vinyl coated expanded metal perforated slabs (61 cm × 76 cm) were placed under drinkers and feeders to minimize soil structure damage. 

Two weeks before starting the second grazing period, a mower was used to homogenize the height of the forage to 15 cm. The biomass was allowed to decompose on site. 

### 2.5. Samplings and Estimations

#### 2.5.1. Soil Sampling

**Figure 7:**
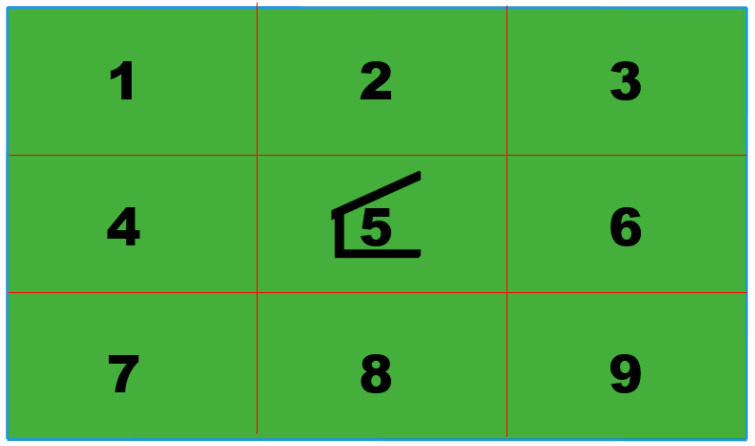
Each paddock was divided into nine equally-sized sections for sampling purposes.

Soil sampling was conducted in December before starting the experiment and in August immediately after animal removal at the end of the second grazing period. For the purposes of soil sampling, each paddock was visually divided into nine equally-sized sections using polyvinyl chloride PVC step-posts along the fence line (Figure 7). From each section of the paddocks, 12 core soil samples were randomly collected at two depths (0 to 15 cm and 15 to 30 cm) using a hand auger (Oakfield 36” LS). These 12 soil core samples were pooled into one composite sample per section and soil depth. Soil samples were kept at 4 C until analyzed at the North Carolina Department of Agriculture and Consumer’s Services (NCDA& CS) soil laboratory where they were analyzed for percent humic matter (HM%), weight volume^−1^ ratio (BD), cation exchange capacity (CEC), percent base saturation (BS%), exchangeable acidity (AC), pH and content of nutrients P, K, Ca, Mg, S, Mn, Zn, Cu, Na and Fe following Melich-3 extraction methodology (Mehlich buffer acidity) [29]. A total of 324 samples were sent to the laboratory, 162 for baseline sampling and 162 following the second grazing period, respectively. In addition to the above mentioned variables, 36 composite samples (18 from the initial sampling and 18 following pig removal) were prepared and analyzed for nitrate nitrogen content [30]. 

#### 2.5.2. Vegetative Cover

During each grazing period, the vegetative cover was recorded weekly in every paddock along 11 transects permanently identified with plastic PVC pipes (1.3 cm diameter) placed along the longest sides of the paddocks. The two exterior transects were located 0.5 m from the paddock fence while the distance between the other transects were 3.0 m for replicates 1 m and 2 m and 3.40 m for replicate 3. A modified step point method was used to identify living vegetation, dead-dormant vegetation and bare soil every other step along the transects [31]. In September, four weeks after the end of the second grazing period, the vegetative ground cover was estimated again along the same transects. Ground cover data analyzed in this study include data from week 8 and 12 of both periods (moments in which all paddocks had been grazed) and data from the last assessment four weeks after ending the second grazing period.

#### 2.5.3. Botanical Composition

In September, four weeks after removing the second batch of pigs, the botanical composition of the paddocks was visually assessed by randomly throwing fifteen quadrats (0.5 m by 0.5 m) in each section of the paddocks [32]. The pasture species present were grouped as tall fescue, crabgrass, other grasses or broad leaves species. 

#### 2.5.4. Nutrients Balance

A simplified nutrients balance estimation was conducted. Feed composition data, feed disappearance and pig weight gain were used to estimate nutrients (N, P and K) balance as differences between inputs to the system and the outputs as pigs’ body components. The coefficient of the retention of the nutrients in the body of pigs used (0.029 kg, 0.0055 kg and 0.0022 kg of N, P and K kg^−1^ body weight, respectively) were obtained from the literature [33,34]. 

### 2.6. Statistical Analysis

Stocking methods were compared by way of analysis of variance/covariance fitting generalized mixed models through the PROC GLIMMIX procedure of SAS 9.4 (SAS Institute Inc., Cary, NC) [35]. Differences between means with significant effects were determined by comparing the least-squares means using the PDIFF statement with a SIMULATE adjustment for multiple comparisons. Repeated measures were tested using a first-order autoregressive structure (AR (1)). Significance was determined at a level of *p* < 0.05 for main factors and *p* < 0.10 for the interactions. Results presented in tables and figures are arithmetic means and standard errors.

The model statements for soil nutrients included fixed effects of stocking methods and soil sampling depths and their interactions and initial values as covariates. Blocks and their interactions were considered as random effects. The sampling sections of the paddocks were included as repeated measures. Differences in ground cover were evaluated by treating stocking methods, grazing periods, their interactions and weeks as fixed effects. Blocks and the interaction blocks × grazing periods were analyzed as random effects. Weeks were considered as repeated measures. Final ground cover (considering week 8 and 12) was evaluated including grazing periods, stocking methods, their interaction and weeks nested within grazing periods as fixed effects. Blocks and their interactions were included as random effects. To test for significant effects of ground cover estimated four weeks after ending the second grazing period, stocking methods were modelled as fixed effects and blocks as random effects. Similarly, the model for botanical composition estimated at the same time included stocking methods, pasture sections and their interactions as fixed effects and blocks as random effects. The section of the paddock was considered as a repeated measure. In the models used to test animal-related variables (final body weight, weight gain, feed disappearance and gain to feed ratio), grazing periods, stocking methods and their interaction were included as fixed effects and blocks were evaluated as random effects. Pig initial weight and sex of the animal were included as covariates in the models for final weight, total weight gain and daily weight gain. No terms were added nor removed from the initial models.

## 3. Results 

### 3.1. Soil Properties

**Figure 8:**
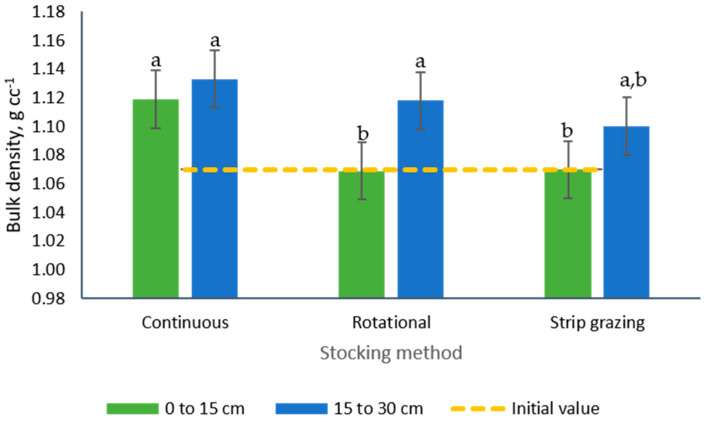
Interaction of stocking method and soil sampling depth on the bulk density of soils from tall fescue paddocks managed with growing-finishing pigs. a, b: means displaying the same letter are not significantly different at the 5% level of probability as indicated by the Multiple Comparisons test—simulate option. Data are the means of three field replicates. Errors bars represent plus or minus one standard error of the means for the interaction.

Interactions between stocking methods and soil sampling depth (Table 2, Figure 8) were observed for bulk density (*p* < 0.0001). While no differences were observed among soil depth for the samples collected from paddocks managed under continuous stocking (average: 1.13 g cc^−1^), the samples collected from rotationally managed paddocks showed greater (+4.7%, 1.12 vs. 1.07g cc^−1^) bulk density in the deeper soil layer (15 to 30 cm). No differences between soil layers were detected among bulk densities in samples from the strip-grazing stocking method (average: 1.9 g cc^−1^).

Differences among samples from different sampling depths (Table 2) were observed for humic matter (*p* = 0.0046), bulk density (*p* ≤ 0.0001) and exchangeable acidity (*p* = 0.0002). In the studied samples, the upper soil layer (0 to 15 cm) presented 46.2% and 11.6% greater values for humic acid and exchangeable acidic, respectively and 2.8% lower bulk density than the values recorded from samples collected deeper in the soil profile (15 to 30 cm).

Similarly, significant effects of the stocking method were observed for soil concentrations of NO_3_^−^—N, P, K, Mn, Zn and Cu, with lower values of these nutrients found in samples from paddocks managed with the rotational stocking method. The soil sampling depth showed significant effects in soil concentrations of NO_3_^−^—N, P, K, S, Mn, Zn, Cu, Na and Fe (Table 3). 

It was estimated that after the two grazing cycles circa 371, 74 and 98 kg ha^−1^ of N, P and K, respectively, were imported into the system via concentrate feed. Similarly, approximately 168, 32 and 12 kg ha^−1^ of N, P and K respectively, were removed as pig body components. The balance, following two pig grazing periods, resulted in the deposition to the paddocks of 204, 42 and 85 kg ha^−1^ of N, P and K, respectively (Table 4). 

### 3.2. Vegetation

**Figure 9:**
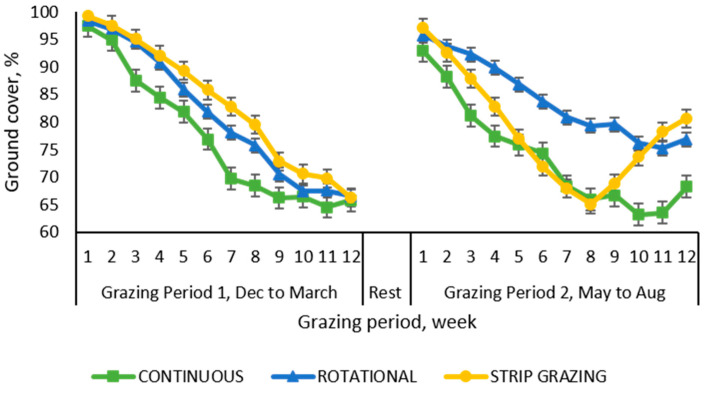
Weekly evolution of the vegetative ground cover in tall fescue paddocks managed with growing-finishing pigs under three different stocking methods. Data are the means of three field replicates. Errors bars represent plus or minus one standard error of the means.

The ground cover of the paddocks declined with time across all treatments (Figure 9). The tendencies in the evolution of the vegetative ground cover were similar for the rotational and strip-grazing stocking, which demonstrated a gradual decline while paddocks managed with the continuous system showed a more marked decrease. By the end of the first grazing period the cover reached levels of 73% on average for all plots, with the plots managed continuously showing 11% less cover than paddocks managed under the other two treatments. The rest period between the two grazing periods allowed the vegetation to recover from the stress inflicted by the animals, however the recovery was not complete and it did not reach 100% ground cover. During the second grazing period, the paddocks managed under rotational stocking displayed greater ground cover than the paddocks managed with the other two stocking methods during most of the sampled weeks. However, at the end of this period the ground cover reached on average the level of 74.1%, with the continuous treatment showing 9% less ground cover than the other two stocking methods which were not dissimilar to each other. A sudden downward trend in ground cover was observed for the paddocks managed by strip-grazing until week 8 when a reverse trend was observed and maintained until the end of the study.

The analysis of variance of the ground cover data pertaining to weeks 8 and 12 (moments when all the sections of the paddocks had been grazed) showed significant effects (*p* = 0.0107) for the interaction between grazing periods × stocking methods. In both periods, paddocks managed with the rotational and the strip grazing stocking did not differed and presented more ground cover (23.8% and 20.2% more for grazing periods 1 and 2, respectively) than paddocks managed continuously which presented on average 67% of ground cover.

**Figure 10:**
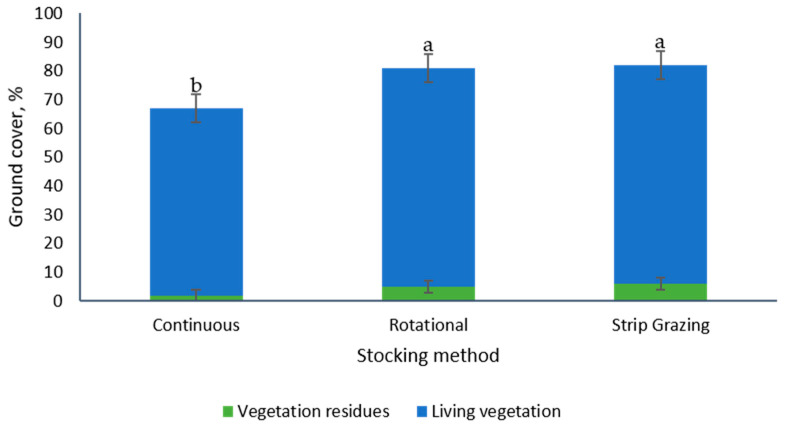
Ground cover (%) in tall fescue paddocks managed with growing-finishing pigs four weeks after the second grazing period. a, b: means displaying the same letter are not significantly different at the 5% level of probability as indicated by the Multiple Comparisons test—simulate option. Data are the means of three field replicates. Errors bars represent plus or minus one standard error of the mean.

The ground cover was also evaluated four weeks following the removal of the animals after the second grazing period. Stocking methods had a significant effect on ground cover (*p* = 0.0064), with greater (+21%) values in paddocks under the rotational and the strip grazing stocking methods than the continuous stocking method (Figure 10). The botanical composition of the paddocks was also estimated at this time, with no effect of the stocking methods on percent of tall fescue (*p* = 0.7248, 59% to 72%), crabgrass (*p* = 0.693, 23% to 37%) and broad leaves species (*p* = 0.8461, 3% to 5%). 

### 3.3. Animal Performance

Initial and final live weight differed among animals used in the two grazing periods. Pigs were heavier at the beginning of the trial during the May to August grazing period (29.1 kg) compared with pigs used in the December to March period (17.5 kg Table 5). Nevertheless, total and daily weight gain were similar among grazing periods. Feed disappearance was greater (+7.46%) during the first grazing period, whereas the gain to feed ratio was greater (+11.1%) during the second one. Animal weight gains were similar in both grazing periods. The stocking method had a significant effect in final weight, total weight gain, daily weight gain, feed disappearance and gain to feed ratio. In general, animals under rotational stocking performed better, showing an 8.8% greater weight gain than the animals on the other two stocking systems.

## 4. Discussion

Stocking methods are techniques that allow the management of grazing animals according to the pasture area and occupation time to reach a determined objective [24]. More than 12 alternative stocking methods derived from continuous or rotational stocking methods have been listed [24]. It was hypothesized that different stocking methods to manage pigs on tall fescue pastures would present different impacts on the soil, vegetation and animal performance. It was expected that the implementation of rotational stocking methods would enhance soil properties within the paddocks, improve ground cover persistence, sustain appropriate animal performance and increase soil nutrient uptake by the forage growing on the pastures. 

### 4.1. Soils Physical-Chemical Properties

Most of the soil variables did not differ significantly among stocking methods, which could be attributed to having managed all the paddocks with the same stocking rate (47 pigs ha^−1^). Another possible reason for the lack of differences could be related to the experiment length. Greater differences could become more evident after longer or more grazing periods. The results showed, however, that pig stocking methods influenced soil bulk density. Lower bulk density was observed from the paddocks managed rotationally or under strip grazing than under continuous grazing. As soil bulk density increases as a result of pig trampling, it could be reasonable to assume that pigs under continuous stocking, being free to use the whole area at will, would tend to concentrate their activity in already-used areas, thus increasing their bulk density. Pigs tend to concentrate in areas adjacent to shelters, feeders and drinkers and patch-perpetuating behavior has been previously observed [15]. Similarly, Bordeaux et al. reported greater bulk density in pastures managed with pigs under a stationary scheme, compared to the bulk density of paddocks where service structures (shelter and water) were moved weekly [18]. Likewise, a study comparing stocking methods for cattle reported that lower bulk density values were recorded in the rotational managed soils [36]. In the present study, pigs under rotational stocking had access to the grazing areas for only one week, although they had permanent access to the service-central area. As the latter represented proportionally a smaller area of the paddock (11.11%) it could have impacted more localized soil areas [37,38]. Conversely, rooting behavior, which has been related to reduction in soil compaction [18,39], could have happened more frequently in the more extensive grazing areas. Additionally, pastures managed under continuous stocking exhibited lower vegetative ground cover than the other two methods. The presence of ground cover could have partially alleviated the direct impact of pig hooves on the soil contributing to a reduction in the soil bulk density of the rotationally stocked paddocks.

Differences were also observed between some of the soil properties in samples collected at different depths. Soil bulk density was greater deeper in the soil, while humic matter concentrations and exchangeable acidity showed higher values in the topsoil layer (0 to 15 cm). These differences could be attributed to the deposition of manure and organic matter to the surface of the ground [15]. The addition of organic matter has also been related with lessened bulk density [40]. 

### 4.2. Soil Nutrients

Soil samples showed differences in their concentrations of NO_3_^−^, P, K, Mn, Zn and Cu among paddocks managed with different stocking methods. Greater concentrations of the above-mentioned soil nutrients were found in paddocks managed under continuous stocking. Differences in soil nutrients among continuous and rotationally stocked pastures have been related to the length of grazing periods [41]. In the present study, the differences could be attributed to the short occupation period (1 week) that would have allowed the forage to rest and recover from animal disturbances in the rotational and strip grazed paddocks and the uptake of some of the nutrients deposited through the manure and feed wastage. Conversely, having unrestricted freedom of movement, pigs in the continuous system could have exerted a greater selective pressure on the vegetation of their preference, thus causing localized overgrazing. Recurrent defoliations and soil disturbance have a negative impact on forage root systems, resulting in a reduction of nutrient uptake [41]. In addition, as a consequence of the weekly movement of feeding (rotational and strip grazing) and watering stations and shelters (strip grazing), the elimination behavior of pigs could have been modified [42], causing a better dispersion of nutrients [41,43] and a greater forage uptake, thus leaving lower concentrations of nutrients in the soil. Conversely, in paddocks under continuous stocking the feeding, drinking and resting areas were kept unchanged for the entire length of the grazing period. In addition, the short grazing periods followed by the rest period could have improved forage nutrient uptake efficiency by supplying nutrients via manure when forages need them for regrowth [44]. Similar results have been obtained in paddocks grazed with cattle where the stocking methods influenced the concentrations of NO_3_^−^, P, K, Mg, Ca and S, with lower concentrations of nutrients recorded in paddocks managed with the shorter occupancy [36].

The concentration of NO_3_^−^, P, K, S, Mn, Zn, Cu, Na and Fe varied among the soil layers with greater values found in the top 0 to 15 cm layer for most of the nutrients but for Fe which presented higher values on the bottom strata of the explored soil profile (15 to 30 cm). Previous studies have reported vertical displacement of soil nutrients [15,36,43]. Grazing pigs deposit dung and urine on the surface of the paddocks. Manure needs to be physically degraded before releasing its components to the soil. However, availability of manure nutrients for plant uptake will be dependent on microbial activity [45] and environmental conditions. It is likely that greater concentrations of nutrients in the upper layer would be a consequence of the manure deposition on the topsoil.

When comparing the initial level of nutrients with those in samples collected following the second pig grazing period (Table 3), changes of different magnitudes were observed. For example, increases in the concentrations of NO_3_^−^-N were observed for both soil layers, the P soil concentrations showed a decrease in the upper soil and an increase in the 15 to 30 cm strata, while K concentrations showed increments for both the upper and bottom soil layers, respectively. Similarly, increases in concentration of inorganic-N forms (53% more NO_3_^−^-N and 75% more NH_4_^+^-N) were reported in paddocks (ryegrass, white and red clover) managed during two 12-week periods with a stocking rate equivalent to 58 pigs ha^−1^ [46]. In the present study, the number of grazing periods evaluated and their length can explain the lack of effects on some of the soil properties. 

Tall fescue, the forage species established on the experimental site, has been described as a cool season perennial forage with good yield potential, drought-resistance, tolerance to close grazing and showing good persistence [47]. Annual dry matter yields (when forage was harvested at 8.5-cm stubble height) have been reported in the range of 6.2 to 9.4 t ha^−1^ yr^−1^ and nutrient uptake potential of 57.3; 10.4; 75.4; 20.9 and 45.1 kg ha^−1^ yr^−1^ of N, P, K, Cu and Zn, respectively [48]. In North Carolina, in soils similar to those where the experiment was conducted, tall fescue have shown the potential to produce up to 5600 kg of forage ha^−1^ yr^−1^ and to remove 240 and 39 kg ha^−1^ yr^−1^ of N and P respectively [49]. If contrasted with the nutrients balance estimation for the systems under study that estimated deposition on average of 204, 42 and 85 kg ha^−1^ of N, P and K (Table 4), respectively, tall fescue pastures have the potential to uptake most of those nutrients. However, in these systems where no forage is removed from the paddocks, the accumulation of nutrients is likely to happen. The implementation of haying is an approach to decrease nutrients loading over time [50].

### 4.3. Vegetation

According to the 2007 North Carolina-Natural Resources Conservation Service recommendation, to minimize erosion in pasture-based pig production systems ground cover needs to be maintained over 75% [51]. Uncontrolled grazing may lead to reduced ground cover [52]. As a consequence of foraging pigs activity, mainly rooting, ground cover endured damage [53]. As in previous studies [14,15,16], the sites where pigs tended to congregate, such as resting, feeding and wallowing areas, showed a greater impact. In the present study, however, at the end of the 12-week grazing periods the ground cover was over 66%, greater than the ground cover (8% to 27%) reported by Kongsted and Jakobsen for pigs grazing (338 m^2^ pig^−1^, 30 pigs ha^−1^) a multispecies pasture including grass—clover and forage herbs for a similar period of time [53] and greater than the final ground cover recorded for bermudagrass pastures [16]. It is worth noting that grazing management strategies (animal breed, forage species, stocking rate, stocking methods and supplemental feed provision) varied between the above mentioned studies.

Greater ground cover was recorded in paddocks managed with rotational and strip grazing methods in comparison with the values obtained under continuous stocking. This advantage may be explained by the short occupation period (1 week) and the rest period (7 weeks) in both rotational stocking methods, while the action of the pigs was uninterrupted under continuous stocking, which could have led to the exhaustion and disappearance of forage plants. Similarities in ground cover among both rotational alternatives could be explained by the impact received by the vegetation under the shelter, drinkers and feeding sites which were disturbed with each weekly movement in the strip grazing method and could be equivalent to the area set to be used as a service area in the rotational method. Corresponding circumstances were described by Bordeaux et al. when comparing the ground cover of sudangrass (*Sorghum bicolor [L.] Moench*) and ryegrass (*Lolium multiflorum*) in pasture-based pig systems managed with stationary or mobile structures [18]. Similarly, the response of tall fescue paddock grazed with beef cattle was affected by stocking method, with paddocks managed rotationally showing greater ground cover [54].

Paddocks under different stocking methods presented similar botanical composition, with tall fescue maintaining its position as the dominant species (66% of the species present in the paddocks). Similarly, Michalk asserted that to maintain the competitive position in a pasture, the dominant species (in this case tall fescue) needs to represent at least the 60% of the biomass [55]. Nevertheless, the emergence of species that were not present at the beginning of the study were observed across stocking methods. Pigs grazing and rooting behaviors could have impacted the vegetation, leaving bare soil patches for other species to colonize [52,56]. Seeds of annual opportunistic species such as crabgrass (*Digitaria sanguinalis*) could have been waiting for ideal conditions to sprout. At the moment of the evaluation, crabgrass was the second most important species present in the paddocks (30%), whereas ragweed (*Ambrosia artemisiifolia*) dominated the broad leaves group which represented 4% of the species present in the paddocks. Differences in botanical composition of paddocks grazed by pigs have been previously reported [57]. Those differences could be partly attributed to the selective grazing behavior exerted by pigs on pasture which tend to select certain plants and moreover parts within a plant [58], leaving the less preferred species to dominate the stand [52]. Similarly, replacement of the dominant plant species by annual vegetation due to the impact of pigs rooting on species composition have been reported previously [56]. Accordingly, the loss of ground cover would be the initial effect of disturbance by pigs, followed by the apparition of annual species that would take advantage not only of the physical removal of competitors but also of the nutrients available in the soil [56].

The values presented across treatments for both ground cover (61%, 81% and 81% for continuous, rotational and strip-grazing method, respectively) and botanical composition (66% fescue, 30% crabgrass and 4% broad leaves) four weeks following animal removal from the paddocks after the second grazing period, could indicate that the pasture system possesses resilience, a trait that can promote a quick recovery from the damage inflicted by the pigs.

### 4.4. Animal Performance

Feed disappearance was 8.06% greater during the first grazing period (December to March, winter) than in the second one (May to August, summer). The effect of environmental temperature on feed intake is known and taken into account for energy requirements (NRC, FEDNA). Voluntary feed intake increased when the temperature was under the lower limit of the comfort zone [59,60] and decreased when the environmental temperature surpassed the upper limit of the temperature comfort zone which decreases as pig age [5,61]. Other reason for this higher winter feed intake is the lower pasture availability in winter respect to spring.

Higher dry matter intake is expected in pasture-based pigs (15% more than indoor-managed pigs) to balance the increased energy requirements (for thermoregulation and exercise) [62,63]. Therefore, feed disappearance values registered in this study (2.01 and 1.86 kg pig d^−1^ for winter and summer, respectively) could seem low when compared with intake values recorded by other researchers, suggesting that tall fescue could have made a contribution to the nutrition of the pigs [63,64]. Intake of forages in grazing pigs represents between 10 and 20% of the total dry matter (DM) intake [65,66]. Greater feed intake values (2.9 kg pig d^−1^) than those recorded in this study have been reported for pigs managed in a daily strip grazing system [60] or for weekly rotated pigs (3.15 kg DM pig d^−1^) [46]. These last authors recorded intake of grass of 0.26 kg DM pig d^−1^ [46]. Jakobsen et al. reported intakes values of alfalfa in the range of 0.33 to 0.47 kg DM pig d^−1^ [65] with similar feed intakes to those registered in this study (2.2 kg DM pig d^−1^).

Previous studies reported no differences in supplementary feed intake (average: 2.4 kg pig d^−1^) among pigs grazing white clover under different stocking methods [64]. Similarly, no differences were observed in the grazing behavior or pasture intake of European wild boar managed either in a continuous or in a rotational grazing system [23]. The feed efficiency values measured in the present study were greater (+8%) in pigs from rotationally managed paddocks than for pigs continuously stocked, while the values obtained from pigs in the strip-grazing treatment were intermediate. Similar values have been registered for Cinta Senese pigs [67], while lower feed efficiency (0.23 kg kg^−1^) has been reported for grazing pigs in Uruguay [46].

It is important to note the potential exposure of the pigs included in this study to fescue toxicosis, which can cause decreased feed intake [68]. It has also been reported that the effects are more evident during summer than during winter [68]. The tall fescue pastures used in this study were not tested for the presence of the fungal endophyte species (*Epichloë coenophiala* Bacon and Schardl.) involved in the symbiotic relationship with the forage [69]. 

Differences in pigs final live weight and total and daily weight gain were found among pigs reared in the different stocking methods, with pigs in rotational paddocks showing greater (8.8%) weight gains that pigs under continuous or strip-grazing stocking. Pasture rotationally stocked had a higher forage quality as reported previously for mixed warm and cool-season grass pastures managed with horses showing differences in concentrations of fiber (ADF, NDF and lignin), water soluble carbohydrates and sugar and digestible energy [70]. So, this higher intake and feed efficiency in rotational grazing produced the highest weight gain. However, this doesn’t happen in strip-grazing stocking pigs, who showed lower feed intake and intermediate feed efficiency. The weekly movement of shelter and drinking structures in the strip grazing paddocks could have produced a lower water consumption and as a consequence lower feed intake. Moreover, the change of all these structures could have disrupted the daily routine of this group of pigs and potentially imposed some sort of stress on them [5,71]. Contrary to these results, no differences in animal weight gain between pigs grazing white clover managed continuously, alternately or rotationally were reported in Brazil [64]. Although pigs were offered the same supplemental feed ad libitum, feed disappearance was similar between continuously and rotationally managed pigs and greater (8.5%) than the values observed for strip-grazed pigs. It had been expected that pigs in the two alternative stocking methods would express increased exploratory and foraging activities as a consequence of their weekly access to new sections of the paddocks [72]. This increased activity could be related to greater energy expenditure due to more exercise and could partially explain the lower weight gain showed by pigs under strip-grazing management.

The total weight gain was similar in both grazing periods and averaged 61.5 kg pig^−1^, the same weight gain figure reported for rotationally stocked pigs during 12 weeks in Uruguay [46]. The daily weight gain recorded in this study was in the range of values reported for Iberico pigs (0.74 to 0.78 kg pig d^−1^) [73] and similar to the values registered for Cinta Senese pigs (0.71 to 0.76 kg pig d^−1^) [67] or for pigs managed in a weekly rotation system (0.73 kg d^−1^) [59] but are lower than those obtained in strip grazing managed pigs (0.90 kg pig d^−1^) [60]. The forage species in the pasture has effects on weight gain. In grass pastures with ad libitum access to supplemental feed, daily gains in the range of 0.59 to 0.88 kg d^−1^ have been reported, whereas for alfalfa pastures the figures were 0.74 to 0.90 kg pig d^−1^ [65].

In this study, it is hard to ascertain the magnitude of the contribution of tall fescue to pig diets, if any. Pigs had ad libitum access to supplemental feed, a factor that has been shown to reduce forage intake [74]. Nevertheless, the possibility of pigs being subjected to toxicity by ingesting endophyte infested fescue cannot be ruled out. Fescue toxicity has been reported to negatively affect animal performance in different livestock species including pigs [68]. 

A practical disadvantage encountered with the strip grazing stocking method was the workload involved in the weekly rotation of the shelters, feeders and drinking structures, which could limit the adoption of this alternative management. 

## 5. Conclusions

The implementation of best management practices is the initial step to reach sustainability with the purpose being to reduce the impact of animal production on the environment and to optimize the efficiency, productivity and profitability of pasture-based systems. The results of this study denoted the positive influence of the use of rotational stocking methods in pastured pig systems in terms of soil bulk density, soil concentrations of NO_3_^−^, P, K, Mn, Zn and Cu and vegetation ground cover maintenance. Animal performance, however, increased only under rotational stocking.

The appearance of spontaneous, less desirable vegetation species in the pastures as a result of pig grazing behavior could lead to reduced pasture quality, underlying the benefits of implementing appropriate pasture management practices that consider conservation-oriented stocking rates and stocking methods. Conversely, these less desirable plant species play an important role in biodiversity conservation and as ground cover.

As the impacts of pigs grazing may only be noticed in the long term, longer studies are needed comparing the effects of different stocking rates and stocking methods on forage persistence and soil properties in pasture-based pig production systems. Nevertheless, these short term studies do show that with an appropriate management it is possible to maintain ground cover and they help to develop expected amounts of nutrient loading of pastures. Because very little vegetation is removed from the sites during pig grazing, nutrient loading will be quicker than in other livestock grazing systems. 

As the strip-grazing stocking method showed potential for enhancement of the environmental performance of pasture-based pig systems, it would be interesting to explore alternatives to improve the ease of performing the rotation of shelters and other structures, which could empower shorter occupation periods. In addition, the impact of scaling up the systems (larger paddocks and herd size) on animal behavior, animal performance and on environmental variables should be examined.

## Figures and Tables

**Table 1:** Stocking methods under evaluation.

Grazing Period	Stocking Method	Area in Use (%)	Stocking Density (m^2^ pig^−1^)
Weeks 1 to 8	Continuous	100	211
	Rotational	22.2 ^1^	47
	Strip	12.5	26
Weeks 9 to 12	Continuous	100	211
	Rotational	33.3 ^2^	70
	Strip	25 ^3^	53

^1^ 11.1% service/central area +11.1% grazing paddock. ^2^ 11.1% service/central area +11.1% grazing paddock +11.1% grazing paddock. ^3^ 2 grazing strips (12.5% + 12.5%).

**Table 2:** Effect of stocking methods and soil sampling depths on the chemical-physical soil properties of tall fescue paddocks managed with growing-finishing pigs.

	HM	BD	CEC	BS	AC	pH
	(%)	(g cc^−1^)	(Meq 100 cc^−1^)	(%)	(Meq 100 cc^−1^)	
Initial value						
0 to 15 cm	0.36	1.04	6.33	79.04	1.28	5.61
15 to 30 cm	0.28	1.1	5.13	79.49	1.0	5.6
SE	0.01	0.01	0.12	0.81	0.04	0.05
Stocking method SM				
Continuous	0.33	1.12 a	5.89	79.54	1.13	5.56
Rotational	0.32	1.09 b	5.77	80.9	1.13	5.61
Strip Grazing	0.32	1.08 b	6.05	78.42	1.29	5.64
SE	0.02	0.02	0.19	2.59	0.18	0.07
Soil depth SD					
0 to 15 cm	0.38 a	1.08 b	5.93	79.69	1.25 a	5.57
15 to 30 cm	0.26 b	1.11 a	5.87	79.55	1.12 b	5.64
SE	0.02	0.01	0.17	2.2	0.18	0.04
Stocking method SM						
*p*	0.6877	0.0400	0.2415	0.6152	0.2703	0.6879
Soil depth SD					
*p*	0.0046	<0.0001	0.5411	0.7865	0.0002	0.1319
SM × SD						
*p*	0.2454	<0.0001	0.6382	0.7641	0.6223	0.6380

*n* = 162; HM: Humic matter; BD: soil density estimated as weight volume^−1^ ratio; CEC: Cationic exchange capacity; BS: Base saturation; AC: exchangeable acidity; pH. *p*: Probability, SE: standard error; SM: Stocking method; SD: Soil sampling depth. a, b, c: means in a column followed by a common letter are not significantly different by the *t*-test at the 5% level of significance. Initial values: nutrients in soil from samples collected from each paddock before grazing with pigs.

**Table 3:** Soil nutrients in tall fescue paddocks managed with growing-finishing pigs during two twelve-week grazing periods.

	NO_3_^2^	P	K	Ca	Mg	S	Mn	Zn	Cu	Na	Fe
	(mg kg^−1^)										
Initial value ^1^											
0 to 15 cm	3.44	51.8	55.2	698.1	172.1	13.4	62	5.3	2.5	18.5	606
15 to 30 cm	2.33	22.2	26.8	602.6	128.2	10.2	47.7	2.5	1.5	16	654.6
SE	0.8	2	1.5	17.9	5.8	0.4	1.9	0.1	0.1	0.6	11.6
Stocking method SM											
Continuous	21.8 a	49.5 a	92.4 a	643.4	152.3	13.7	45.9 a	4.3 a	1.9 a	22.6	693.9
Rotational	16.9 b	41.0 b	72.4 b	650.3	155.3	13.6	39.3 b	3.6 b	1.7 b	25.2	660.9
Strip Grazing	18.1 a,b	40.1 b	75.9 b	655.1	157.1	13.1	45.8 a,b	3.8 b	1.8 a,b	25.2	670.7
SE	1.5	2.2	4.6	20.8	5.6	0.8	2.7	0.3	0.1	2.3	33.6
Soil depth SD											
0 to 15 cm	25.2 a	50.9 a	109.7 a	654.2	150.3	15.0 a	45.3 a	4.7 a	2.1 a	26.1 a	657.2 b
15 to 30 cm	12.8 b	36.2 b	50.8 b	645.1	159.6	11.9 b	42.1 b	3.1 b	1.5 b	22.5 b	692.9 a
SE	1	2.1	4.6	14.1	6.1	0.8	1.8	0.3	0.1	1.4	27.4
Stocking method SM											
*p*	0.0627	0.0052	0.0037	0.9195	0.6309	0.6236	0.0701	0.0016	0.0077	0.6879	0.6468
Soil depth SD											
*p*	<0.0001	<0.0001	<0.0001	0.4807	0.2700	<0.0001	<0.0001	<0.0001	0.0095	0.0003	0.0007
SM × SD											
*p*	0.8020	0.4003	0.8689	0.3080	0.7000	0.9907	0.5556	0.1329	0.1168	0.2527	0.9256

*n* = 162; NO_3_^−^ nitrate, P: Phosphorus, K: potassium, Ca: calcium, Mg: magnesium, S: Sulphur, Mn: manganese, Zn: zinc, Cu: copper, Na: sodium, Fe: Iron. P: Probability, SE: standard error; SM: Stocking method; SD: Soil sampling depth. ^1^ Initial values: nutrients in soil from composite samples collected from each paddock before grazing with pigs, *n* = 18; ^2^: composite soil samples collected from each paddock after grazing with pigs. a, b: means in a column followed by a common letter are not significantly different at the 5% level of probability as indicated by the Multiple Comparisons test—simulate option.

**Table 4:** Balance of Nitrogen, Phosphorus and Potassium (kg ha^−1^), in tall fescue paddocks managed with growing-finishing pigs.

		N kg ha^−1^			P kg ha^−1^			K kg ha^−1^		
		Continuous	Rotational	Strip Grazing	Continuous	Rotational	Strip Grazing	Continuous	Rotational	Strip Grazing
**Input**	**December to March**	200	196	188	32	31	30	49	49	47
	**May to August**	181	184	163	44	44	39	50	51	45
	**TOTAL**	381	380	351	76	76	69	99	100	92
**Output**	**December to March**	79	89	81	15	17	15	6	7	6
	**May to August**	85	88	80	16	17	15	6	7	6
	**TOTAL**	165	177	161	31	34	31	12	13	12
**Excreted**	**December to March**	121	107	107	17	14	14	43	43	41
	**May to August**	96	95	83	28	28	24	43	44	39
	**TOTAL**	216	202	190	45	42	39	87	87	80

N: Nitrogen; P: Phosphorus; K: Potassium; Input: estimated as a function of amount of nutrients in feed and feed disappearance; Output: estimated amount of nutrients (N, P and K) in the body of grower to finisher pigs (30 to 100 kg). The coefficients of retention used 0.029, 0.0055 and 0.0022 kg of N, P and K kg^−1^ body weight, were obtained from the literature [28,29]. The stocking rate was equivalent to 47 pigs ha^−1^.

**Table 5:** Effect of stocking method on performance of growing-finishing pigs reared in tall fescue paddocks.

	Live Weight		Weight Gain		Feed	
	Initial	Final	Total	Daily	Disappearance	Gain to feed
	(kg pig^−1^)	(kg pig^−1^)	(kg pig^−1^)	(kg pig^−1^ d^−1^)	(kg pig^−1^ d^−1^)	(kg kg^−1^)
Grazing period GP						
December to March	17.5 b	78.66 b	60.9	0.73	2.01 a	0.36 b
May to August	29.14 a	90.98 a	62.09	0.74	1.86 b	0.40 a
SE	0.59	1.37	1.66	0.02	0.03	0.01
Stocking method SM						
Continuous	23.18	83.56 a,b	60.37 b	0.72 b	1.99 a	0.37 b
Rotational	23.30	88.29 a	64.99 a	0.77 a	1.98 a	0.40 a
Strip Grazing	23.47	82.60 b	59.13 b	0.70 b	1.83 b	0.39 a,b
SE	0.72	1.68	1.54	0.02	0.04	0.01
Grazing period GP						
*p*	<0.0001	<0.0001	0.6717	0.6717	0.0100	0.0040
Stocking method SM						
*p*	0.9585	0.0414	0.0208	0.0208	0.0404	0.0714
GP × SM						
*p*	0.9975	0.4937	0.3707	0.3707	0.5723	0.3966

a, b: means in a column followed by a common letter are not significantly different at the 5% level of probability as indicated by the Multiple Comparisons test—simulate option.
